# In-hospital informal caregivers' needs as perceived by themselves and by the nursing staff in Northern Greece: A descriptive study

**DOI:** 10.1186/1472-6955-10-19

**Published:** 2011-10-07

**Authors:** Maria Lavdaniti, Vasilios Raftopoulos, Markos Sgantzos, Maria Psychogiou, Tsaloglidou Areti, Charikleia Georgiadou, Ismini Serpanou, Despina Sapountzi-Krepia

**Affiliations:** 1 Department of Nursing, Alexander Technological Educational Institute of Thessaloniki, Greece; 2 Nursing Department, Cyprus University of Technology, Mediterranean Research Centre for Public Health and Quality of Care, 215 Paleos dromos Lefkosias-Lemesou, Strovolos, 2029, Nicosia, Cyprus; 3 Department of Anatomy, Medical School, University of Thessaly, BIOPOLIS, 41110 Larissa, Greece; 4 Department of Nursing Science, University of Kuopio, PO Box 1456, TK 541 01, Thessaloniki, Greece, Finland; 5 A.H.E.P.A Hospital, PO Box 1456, TK 541 01, Thessaloniki, Greece; 6 G. Papanicolaou Hospital, Exohi Thesalonikis 57010, Thessalonica, Greece; 7 Private Practice, 10 Kefalinias street, 16342, Ilioupolis, Athens, Greece; 8 Nursing Department, Frederick University, 7, Y. Frederickou Str. Pallouriotisa, Nicosia, Cyprus

**Keywords:** health education needs, in-hospital care, informal caregivers, informational needs, nursing staff

## Abstract

**Background:**

Informal care is common in many countries, especially in Greece, where families provide care in hospitals. Health education and informational needs are important factors for family members which are often underestimated by nursing staff. The aim of this study was to compare the perceptions of the nurses and the in-hospital informal caregivers about the in-hospital informal caregivers' knowledge and informational needs, as well as the factors that influence these perceptions.

**Methods:**

This was a non-experimental descriptive study conducted in three general hospitals in Greece. The sample consisted of 320 nurses and 370 in-hospital informal caregivers who completed questionnaires. Descriptive statistics were analyzed using t-tests; group comparisons were conducted using ANOVA.

**Results:**

The score of the questionnaire for health education and informational needs was significantly greater for informal caregivers (57.1 ± 6.9 and 26.6 ± 2.8) than for nurses (53.4 ± 5.7 and 22.4 ± 3.1) (p < 0.001). For the nursing staff, the factors that influence the informational needs of patients' caregivers were *level of education *and *working experience*, while for the caregivers the *level of education *was independently associated with the score for the health education needs. Finally, *age, marital status*, and *level of education *of informal caregivers' were independently associated with informational needs.

**Conclusions:**

The in-hospital informal caregivers perceived that they have more educational and informational needs than the nurses did. The findings of this study also show that the nursing staff has to identify the needs of in-hospital informal caregivers in order to be able to meet these needs.

## Background

Informal care is defined as "a non-market composite commodity consisting of heterogeneous parts produced by one or more members of the social environment" [1]. Hospitalized patients are frequently facing problems with their self-care and as a result they need assistance [2]. In Greek hospitals, the nursing staff assists patients; however, the provision of help by in-hospital informal caregivers is a common phenomenon [3]. Family has a central role in maintaining the health status and providing informal health care, and is critical in helping its members to manage with illness [4,5], as well as in assisting in the recovery and rehabilitation process [5-7]. As stated in the literature, family members usually spend a considerable amount of time in the hospital with their hospitalized relatives [8], and these family members have identifiable needs for care [9].

Need is defined as "an internal tension resulting from a change in some state of affairs. This tension is manifested in goal-oriented behavior, which will continue until the tension is relieved and the need is satisfied" [10,11]. The definition of family needs as Leske stated is "a requirement that, if unmet, produces distress" [12].

Family members frequently visit their relatives at the hospital [2,3]. The illness effects the rest of the members and causes changes in the life of the entire family [8]. Various feelings and emotions are experienced, including helplessness, powerlessness, stress, worry, fear, and anxiety [13]. The informational and educational needs of family members were about the progress of patient health, treatment, nursing care and general care that is provided in the hospital [14].

Earlier studies in Greece support that families participate in the care of their hospitalized relatives providing assistance with personal hygiene, feeding, making beds, toileting, bathing, and helping taking oral medications [3,14,15]. In Greece the prevalence of in-hospital informal care is very high. This phenomenon started in the beginning of the 1980s, with the introduction of an open visiting hours policy [3]. Family members were forced, unofficially, to stay at patients' bedsides for many hours to assist with their care [15]. According to the Greek nursing literature this phenomenon is highly correlated with the nursing staff shortage and specific cultural influences on care provision patterns [3,14,15]. Several studies assessing informal caregivers' informational and education needs [16-18] have been published in the nursing literature. Richter and Peu [18] argue that the informal caregivers' education needs are mostly concentrated on health promotion and disease prevention activities, while Beaver and Witham [16] stressed that the priority in informational needs for caregivers of women treated for breast cancer are related to cure, spread of disease, and treatment. Kosco and Warren [19] reported that informational needs pertaining to patients' conditions and to the procedures being performed continue to be a major priority for families. A recent study showed that most of the informal caregivers of persons with dementia reported that they need additional information and advice [20].

Many studies have examined nurses' and family members' perceptions concerning the family's or significant others' needs, especially in critically ill patients or in intensive care units, [21-24] as well as in other patient groups [25,26]. Some studies have documented that family members gave higher scores to the questions that concerned their needs than the nurses themselves [23,24]. However, a limitation of the studies that assessed the needs of informal caregivers was their small samples [16,17,22]. Some studies have used specific scales to measure the perceptions of family's care giving and nurses [17,22,24] and others have both a qualitative and a quantitative design [16,18].

Fewer studies have examined the factors that influence families' and nurses' perceptions of family needs [27-29]. It has been reported that age, sex, socioeconomic status and educational level of family members do not affect their informational needs [27,28]. Verhaeghe et al. [29] reported that women in general, with the exception of the need for information, report more needs than men, that more-educated people have fewer needs than the less-educated, and that the need for support is affected by educational status. The relationships between the factors that influence the perceptions have not been examined systematically.

Although the body of evidence is growing regarding the perceptions of informal caregivers and nurses concerning caregivers' needs, little is known about this issue in Greece. Despite an increasing interest for in-hospital informal care among Greek nurses, informal in-hospital caregivers' informational and educational needs are often underestimated and not recognized by nurses, although these are often informally reported.

The objective of this study was to assess the nursing staff's and informal caregivers' perceptions about the health education and informational needs of in hospital informal caregivers. The specific aims of the study were the following:

a) to compare nurses' and family members' perceptions about the caregivers health education needs,

b) to compare nurses' and family members' perceptions about the caregivers informational needs, and

c) to determine what factors influence the nurses' and family members' perceptions about the caregivers health education and informational needs.

## Methods

### Design and sample

In 2008, a non-experimental descriptive study was conducted in three large general hospitals for patients with chronic diseases, in a major city in Greece. A convenience sample of 320 nurses working in medical wards and 370 informal caregivers acting as in-hospital caregivers for their hospitalized relatives were recruited. The inclusion criteria for nurses and informal caregivers were the following: 1) willingness to participate in the study 2) ability to speak and read Greek 3) age 18 years or older and 4) working in the same hospital units as patients and informal caregivers.

### Data collection

Ethical approval regarding the study protocol was obtained from both the Alexander Technological Educational Institute of Thessaloniki and the hospitals' administration committees. A member of the research team approached all potential participants who fulfilled the eligibility criteria. The aim of the study was explained and the participants were asked whether they were willing to participate in the study. The participants had the opportunity to ask questions about the study and those who agreed to participate were asked to provide oral informed consent. The study sample comprised of 370 informal caregivers and 320 nurses. The response rates were 85.5% and 94.5% respectively. Thirteen participants failed to complete some items of the questionnaire and were excluded.

### Instrument

A specific questionnaire written in Greek, which has been validated and used in prior Greek studies was used, to explore the nurses' and caregivers' opinions on the health education and informational needs of caregivers of patients acting as in hospital caregivers [3,15]. The questionnaire was developed by Sapountzi-Krepia et al [15] and is called In-Hospital Informal Care Questionnaire Acute Care (IHICQAC) (Additional File 1). The first part of the questionnaire contained questions for eliciting information on demographic, social, and working characteristics of the subjects. The second part contained a scale of 13 questions exploring the nursing staff's and in-hospital informal caregivers' opinions on the latter's health education needs. A five-point Likert scale of 13 questions was used which ranged from 1: "very strongly disagree" to 5: "very strongly agree". The scores of the items in this particular scale were added creating a summated scale [28]. The total score for health education needs could vary from 13 to 65 points. Higher scores indicate that nursing staff and in-hospital informal caregivers perceive that informal caregivers have higher health education needs. The third part contained a five-point Likert scale of six questions about nursing staff's and in-hospital informal caregivers' opinions regarding the informational needs of the in-hospital informal caregivers. Possible scores for this scale range from 6 to 30 points. Higher scores indicate that nursing staff and in-hospital informal caregivers' perceive that informal caregivers have higher informational needs.

Face and content validity of the instrument was assessed from a panel of experts in the content area [30], specifically by a general practitioner and two nurses who were recruited in order to review the questionnaire. The development of the questionnaire was based on the relevant literature in combination with the researchers' experiences of the health education and the informational needs of patients' caregivers who act as in hospital caregivers. After many discussions and consultations with other researchers regarding the aims and the objectives of the study, it was decided that only questions related to the health education and to the informational needs of patient's family should be included in the questionnaire.

The content validity of the questionnaire was determined by a general practitioner and two nurses who were called in order to review the questionnaire. The reviewers examined the ability of the questionnaire to extract sufficient information and finally they proposed which questions should be included or eliminated. They also advised the researchers on issues regarding the wording and t discussed whether or not the questions were comprehensible and culturally acceptable. The questionnaire was revised in accordance with the reviewers' suggestions and afterwards was checked one more time. The final format and content of the questionnaire was agreed upon by all professionals involved in the study. Before the completion of the questionnaire, a pilot study was undertaken. In the pilot study purposive sample of patients' caregivers and nurses was used in order that they might give a better answer to the research question. The participants of the pilot study also contributed to the change and the correction of some questions and were not involved in the study sample.

Cronbach's alpha in the present sample for health education and informational needs questionnaire ranged from 0.81 to 0.92 for both two populations.

### Data analysis

Quantitative variables are presented with absolute and relative frequencies. For the comparison of mean total scores concerning health education needs and informational needs between two groups, student's t-tests were used, while for the comparison of the aforementioned scores between three or more groups analysis of variance (ANOVA) was used. If the result of analysis of variance was significant, Bonferroni correction test was also used to control the multiple variables. In order to explore the factors independently associated with the total score for health education needs and informational needs, linear regression analyses were performed with a stepwise method both for nurses and caregivers [31]. All p-values reported are two-tailed, with the statistical significance set at 0.05. Analyses were conducted using SPSS statistical software (version 15.0).

## Results

The mean age of the nurses was 36.7 ± 7.2 years and of in-hospital informal caregivers was 47.3 ± 11.3 years. The majority of the nurses (n = 287, 90%) were women, and 66.3% (n = 203) were married. The majority of the in-hospital informal caregivers (n = 315, 85.1%) were females and married (n = 291, 82.4%). One hundred forty four caregivers (40.4%) were secondary education graduates and one hundred thirty seven (37.0%) were wives/husbands of the patient. The social and demographic characteristics of the sample are presented in Table 1.

**Table 1:** Demographic characteristic of the nursing staff and caregivers

Nursing staff	N	%	Caregivers	N	%
**GENDER**			**GENDER**		
Male	32	10.0	Male	55	14.9
Female	287	90.0	Female	315	85.1
**AGE**			**AGE**		
<34	93	33.9	<42	116	33.5
34-39	90	32.8	42-52	122	35.3
>39	91	33.2	>52	108	31.2
**MARITAL STATUS**			**MARITAL STATUS**		
Single	79	25.8	Single	33	9.3
Married	203	66.3	Married	291	82.4
Widower-widow/Separated/Divorced	24	7.8	Widower-widow/Separated/Divorced	29	8.2
**EDUCATION**			**EDUCATION**		
Secondary education	148	46.5	Primary	83	23.3
University/Technological Educational Institute/Master studies	170	53.5	Secondary	144	40.4
**WORK EXPERIENCE (YEARS)**			Tertiary	129	36.2
<10	110	35.9	**RELATION TO THE PATIENT**		
10-16	94	30.7	Husband/Wife	137	37.0
>16	102	33.3	Mother/Father	87	23.5
**POSITION**			Brother/Sister	32	8.6
Head nurse or replacing the head nurse	30	9.6	Daughter/Son	68	18.4
Nurse	167	53.5	Daughter in law/Son in law	26	7.0
Assistant/vocational nurse	115	36.9	Other	20	5.4

The results of the comparison between nurses' and family members' perceptions about the caregivers' health education and informational needs are shown in Table 2 and 3, including the mean total scores for health education needs and informational needs according to the nursing staff's characteristics and the caregivers' characteristics. The mean total scores for health education needs was 53.4 ± 5.7 (range: 50 to 57) for nurses and 57.1 ± 6.9 (range: 52 to 63) for the in-hospital informal caregivers (p < 0.001). The mean total score for the informational needs was 22.4 ± 3.1 (range: 20 to 24) for nurses and significantly lower (mean: 26.6 ± 2.8; range: 24 to 29) compared to in-hospital informal caregivers (p < 0.001).

**Table 2:** Mean total score for health education needs according to the nurses' and the caregivers' characteristics

NURSING STAFF'S CHARACTERISTICS	Score for health education needs			CAREGIVERS' CHARACTERISTICS	Score for health education needs		
	Mean	SD	P (student's t-test)		Mean	SD	P(student's t-test)
**GENDER**				**GENDER**			
Male	54.0	5.3	0.485	Male	56.7	5.3	0.601
Female	53.3	5.7		Female	57.2	7.2	
**AGE**				**AGE**			
<34	53.1	4.8	0.492*	<42	55.7	8.8	0.070*
34-39	54.0	5.8		42-52	57.5	5.8	
>39	53.9	5.7		>52	57.6	5.7	
**MARITAL STATUS**				**MARITAL STATUS**			
Single	53.5	6.2	0.926*	Single	54.9	8.9	0.140*
Married	53.3	5.5		Married	57.4	6.8	
Widower/widow-Separated-Divorced	53.5	5.9		Widower/widow-Separated-Divorced	58.0	5.1	
**EDUCATION**				**EDUCATION**			
Secondary education	53.2	5.3	0.586	Primary	57.3	5.9	<0.001*
University/T.E.I./Master level studies	53.5	6.0		Secondary	55.6	7.6	
**WORK EXPERIENCE (in years)**				Tertiary	59.1	6.2	
<10	52.8	5.8	0.534*	**RELATION TO PATIENT**			
10-16	53.6	5.9		Husband/Wife	56.4	7.6	0.076*
>16	53.6	5.5		Mother/Father	58.2	6.6	
**POSITION**				Brother/sister	58.8	4.7	
Head nurse or replacing head nurse	52.8	6.9	0.767*	Daughter/Son	55.6	5.6	
Nurse	53.5	5.8		Daughter in law/Son in law	58.9	5.4	
Assistant/Practical nurse	53.1	5.0		Other	56.9	10.3	

*analysis of variance (ANOVA)

**Table 3:** Mean total score informational needs according to the nurses' and the caregivers' characteristics

NURSING STAFF'S CHARACTERISTICS	Score for informational needs			CAREGIVERS' CHARACTERISTICS	Score for informational needs		
	Mean	SD	P (student's t-test)		Mean	SD	P (student's t-test)
**GENDER**				**GENDER**			0.219
Male	21.4	3.1	0.081	Male	26.2	3.4	
Female	22.5	3.1		Female	26.7	2.7	
**AGE**				**AGE**			<0.001*
<34	21.8	2.9	0.006*	<42	25.7	3.0	
34-39	23.2	3.0		42-52	26.7	2.7	
>39	22.6	3.1		>52	27.2	2.6	
**MARITAL STATUS**				**MARITAL STATUS**			0.001*
Single	22.2	3.2	0.568*	Single	24.9	2.1	
Married	22.4	3.1		Married	26.9	2.9	
Widower/widow-Separated-Divorced	23.0	3.6		Widower/widow-Separated-Divorced	26.8	2.3	
**EDUCATION**				**EDUCATION**			<0.001*
Secondary education	21.8	2.9	0.002	Primary	27.6	2.3	
University/T.E.I./Master level studies	22.9	3.2		Secondary	25.9	2.9	
**WORK EXPERIENCE (in years)**				Tertiary	26.9	2.9	
<10	22.0	3.2	0.035*	**RELATION TO PATIENT**			0.051*
10-16	23.1	2.9		Husband/Wife	26.7	3.1	
>16	22.2	3.0		Mother/Father	26.9	2.8	
**POSITION**				Brother/sister	26.8	2.5	
Head nurse or replacing head nurse	23.3	3.4	0.064*	Daughter/Son	25.7	2.8	
Nurse	22.5	3.1		Daughter in law/Son in law	27.4	1.8	
Assistant/Practical nurse	21.9	2.9		Other	26.8	2.3	

*analysis of variance (ANOVA)

After applying the Bonferroni correction, it was found that nurses who were 34-39 years old had significantly higher informational needs compared to those who were < 34 years old (p = 0.004). Furthermore, nurses who were graduates of tertiary education or had completed master level studies had a significantly higher score regarding informational needs compared to the nursing staff who were graduates of secondary education (p = 0.003).

In addition, it was shown that nurses who had 10-16 years of work experience, had significantly higher informational needs compared to the nurses who had less than 10 years of work experience (p = 0.043). The in-hospital informal caregivers who were graduates of tertiary education had a significantly higher score for health education needs than the caregivers who were graduates of secondary education (p < 0.001). In-hospital informal caregivers who were < 42 years old had a significantly lower score about the informational needs than those who were 42-52 years old (p = 0.014) and those who were > 52 years old (p < 0.001). Moreover, in-hospital informal caregivers who were unmarried had a significantly lower score about informational needs compared to those who were married (p < 0.001) and those who were widowers/widows or divorced (p = 0.020). In-hospital informal caregivers who were graduates of secondary education had a significantly lower score about informational needs compared to those who were graduates of tertiary education (p = 0.007) and primary education (p < 0.001).

For determining what factors influence the nurses' and family members' perceptions about the caregivers health education and informational needs a multiple linear regression analysis was conducted. When multiple linear regression analysis (Table 4) was conducted with the total score for informational needs as the dependent variable, it was found that the independent predictors were the educational level and the years of work experience. More specifically, the nurses who were graduates of tertiary education or had completed master level studies, scored 1.29 points higher for informational needs, and those who had 10-16 years of work experience had a score 0.94 points higher than those with less than 10 years of work experience.

**Table 4:** Scores for health education needs and informational needs

	β	S.E.	P
**NURSING STAFF (score for informational needs)**			
			
**Education**			
Secondary education			
University/T.E.I./Master level studies	1.29	0.37	<0.001
**Work Experience (in years)**			
<10			
10-16	0.94	0.43	0.031
>16	0.36	0.42	0.391
			
**CAREGIVERS (score for health education needs)**			
			
**Education**			
Tertiary			
Secondary	-3.77	0.86	<0.001
Primary	-1.61	1.00	0.108
			
**CAREGIVERS (score for informational needs)**			
			
**Age**			
<42			
42-52	0.85	0.39	0.030
>52	0.88	0.42	0.040
**Marital status**			
Single			
Widower-widow/Separated/Divorced	1.63	0.74	0.029
Married	1.49	0.54	0.006
**Education**			
Tertiary			
Secondary	-1.01	0.35	0.004
Primary	0.22	0.44	0.617

(Regression coefficients (β) and standard errors (S.E.) derived from stepwise multiple linear regression analysis with dependent variables).

Multiple regression analysis indicated that the in-hospital informal caregivers' educational level was independently associated with the score for health education needs. Caregivers who were graduates of secondary education scored 3.77 points lower about health education needs than those with tertiary education. Finally, age, family status, and educational level were independently associated with the score about informational needs. Caregivers who were 42-52 years old had a score higher by 0.85 points on average about informational needs than those who were < 42 years old, and a 0.88 points higher score about informational needs compared to those who were > 52. Caregivers of secondary education had a significantly lower score about informational needs than those of tertiary education.

## Discussion

This study compares nursing staff's and informal caregivers' perceptions about the health education and informational needs of family members in Greece. It contributes to the growing body of evidence regarding informal care giving and provides an important foundation for Greek nurses, as describing the phenomenon of informal care is a fundamental step toward appropriate interventions regarding better quality of care.

The results illustrate that the vast majority of in-hospital informal caregivers were middle-age women. This was an expected outcome based on preceding studies [2,5,32]. Moreover, in Greece, women have traditionally held the role of caring for the family [14], as is the case in almost all societies [3].

In this research educational and informational needs of in-hospital informal caregivers were about patients care, disease, treatment, nutrition, insurance funds, etc. In Greek literature in-hospital informal caregivers spend almost the entire day at their patients' bedside, so it is obvious that they need information and teaching about care-giving techniques [15]. Other studies in Western countries report that informational and educational needs of informal caregivers are about health promotion and treatment [16,18]. This difference is due to the peculiar phenomenon of in-hospital informal care giving observed in Greek hospitals.

An important finding is that informal caregivers perceived they have more educational and informational needs than nurses did. This is consistent with the findings in other studies. Nurses underestimate most of the family needs (informational, assurance, proximity) [23,24,26]. However, when comparing this research to other studies, it is important to note that the use of different questionnaires may provide certain limitations. Therefore, there is a need for further research in Greece using reliable scales, in order to clarify and compare the needs of informal caregivers.

An interesting result is that the nurses who were graduates of tertiary education or had completed master level studies, as well as those with several years of work experience, had higher scores in relation to their informational needs. A possible explanation for this finding is that the more educated nurses are more demanding professionals; they tend to provide better nursing care and therefore demand of themselves to provide higher quality services. The finding regarding the difference of work experience is consistent with the findings of other studies [23,33] which showed that nurses with great clinical experience scored higher regarding the needs of significant others than nurses with less clinical experience.

The informal caregivers' needs were found to be clearly related to the demographic variables studied. Older caregivers have more informational needs suggesting that people need more information as they getting older. Married caregivers were found to score the needs higher than unmarried. Also, it was found that caregivers who have a middle level of education have more needs than less and more educated. This is inconsistent with other literature which supports that the more highly educated people are the fewer needs they express [27,29]. In light of the present data, the authors cannot reach any final conclusion regarding this finding. Further research on this particular issue is necessary in order to provide a clear answer.

### Study limitations

One limitation of this study is that the scale was not validated but checked prior to its implementation. In the future a concurrent administration of an established perception tool needs to be collected. Other limitations of this study include the use of a convenience sample, and that the data collection was performed within a small period of time. The findings of this study are based on the investigation of nurses and family members from hospitals in only one Greek city. Additional research using random sampling from hospitals in other cities should be performed in order to enhance the generalizability of the findings.

Factors such as response bias and social desirability response tendencies make it difficult to generalize the findings. Another limitation is that the researchers didn't compare differences between the hospitals or compare different patients groups. In an effort to minimize the effect of these limitations, the instrument was pilot-tested to check if it is understandable and easily manageable by potential participants.

## Conclusions

The results revealed that there was a gap between the perceptions of nurses and caregivers about the needs of family members. The in hospital informal caregivers perceived that they have more educational and informational needs than the nurses did. For the nursing staff, the factors that influence the informational needs of patients' caregivers were the educational level and the years of working experience, while for the caregivers the educational level was independently associated with the score for the health education needs. Finally, age, marital status, and educational level of informal caregivers' were independently associated with informational needs.

Also, it is important for the nurses to identify the needs of the family members in order to find ways to offer more pertinent information to them. The recognition, discussion, and evaluation of those needs by nurses can lead to increased continuity in nursing care and to more time spent providing information.

Moreover, the findings may be of interest to policy makers and health authorities in order to design new plans for the health care system, aiming to improve the degree of satisfaction of the consumers of Greek National Health system. Further research examining the perception of nurses and family members in specialized clinical areas, such as intensive care units, oncology hospitals and emergency departments in Greece could add very important and useful information to the Greek nursing literature.

## Competing interests

The authors declare that they have no competing interests.

## Authors' contributions

M-L, V-R, M-S, D-SK conceived, designed, acquired the data, coordinated the study, analyzed the data, interpreted the results, wrote and revised the manuscript. C-G was mainly responsible for data collection, and M-P, A-T, I-S were involved in the manuscript preparation. All the authors have approved the final version of the manuscript submitted for publication.

## Pre-publication history

The pre-publication history for this paper can be accessed here:

http://www.biomedcentral.com/1472-6955/10/19/prepub

## Supplementary Material

Additional file 1 **The In-Hospital Informal Care Questionnaire Acute Care Questionnaire (IHICQAC) for caregivers and nurses**. The additional file contains the English version of the In-Hospital Informal Care Questionnaire Acute Care Questionnaire (IHICQAC) for caregivers and nurses Click here for file
