# Supplementary material for: In-hospital informal caregivers' needs as perceived by themselves and by the nursing staff in Northern Greece: A descriptive study
Source: BMC Nurs. 2011 Oct 7;10:19. doi: 10.1186/1472-6955-10-19 (PMC3200151; doi:10.1186/1472-6955-10-19)
Supplement: Additional file 1 — The In-Hospital Informal Care Questionnaire Acute Care Questionnaire (IHICQAC) for caregivers and nurses. The additional file contains the English version of the In-Hospital Informal Care Questionnaire Acute Care Questionnaire (IHICQAC) for caregivers and nurses [file 1472-6955-10-19-S1.DOC]

**QUESTIONNAIRE FOR CAREGIVERS**

**1. GENDER**

1. Male
2. Female

**2. AGE**

**3. MARITAL STATUS**

1. Single
2. Married
3. Widower- widow/Separated/Divorced

**4. EDUCATIONAL LEVEL**

1. Primary
2. Secondary
3. Tertiary

**5. RELATION TO THE PATIENT**

1. Husband/Wife
2. Mother/Father
3. Brother/Sister
4. Daughter/Son
5. Daughter in law/Son in law
6. Other

**6. HEALTH EDUCATION NEEDS**

**Do you believe that it would be useful for the patients’ caregivers to be taught about:**

I. Techniques of patient care (for bathing, hair washing, changing position etc.)?

I strongly agree I agree neutral I disagree I strongly disagree

!_______ ! ______!_____ ________!_____________!

5 4 3 2 1

II. Basic knowledge about the patient’s disease when it is a chronic disease?

I strongly agree I agree neutral I disagree I strongly disagree

!_______ ! ______!_____ ________!_____________!

5 4 3 2 1

III. Prevention of pressure sores?

I strongly agree I agree neutral I disagree I strongly disagree

!_______ ! ______!_____ ________!_____________!

5 4 3 2 1

IV. How to manage complications in emergency situations that maybe the patient could develop in order to be able to provide first aid at home?

I strongly agree I agree neutral I disagree I strongly disagree

!_______ ! ______!_____ ________!_____________!

5 4 3 2 1

V. About the patient’s treatment at home in order to be able to help him/her at home?

I strongly agree I agree neutral I disagree I strongly disagree

!_______ ! ______!_____ ________!_____________!

5 4 3 2 1

VI. About the nutrition and daily fluid consumption that the patient should have at home?

I strongly agree I agree neutral I disagree I strongly disagree

!_______ ! ______!_____ ________!_____________!

5 4 3 2 1

VII. About how they can be effective when they seek help for the patient’s care by public services and N.G.O.s?

I strongly agree I agree neutral I disagree I strongly disagree

!_______ ! ______!_____ ________!_____________!

5 4 3 2 1

VIII. About how they can be effective when they seek help for the patient’s care by the extended family?

I strongly agree I agree neutral I disagree I strongly disagree

!_______ ! ______!_____ ________!_____________!

5 4 3 2 1

IX. About basic knowledge about the patient’s emotional needs and the possible changes in behavior that the disease might cause?

I strongly agree I agree neutral I disagree I strongly disagree

!_______ ! ______!_____ ________!_____________!

5 4 3 2 1

X. About how to deal with the stress that they might face due to their increased responsibility for the patient’s care?

I strongly agree I agree neutral I disagree I strongly disagree

!_______ ! ______!_____ ________!_____________!

5 4 3 2 1

XI. About (infection control) how to avoid spreading infections when they deal with the patient’s care?

I strongly agree I agree neutral I disagree I strongly disagree

!_______ ! ______!_____ ________!_____________!

5 4 3 2 1

XII. Do you consider that the hospitals have a responsibility to offer to the caregivers’ educational programmes about caring for patients with chronic health problems?

I strongly agree I agree neutral I disagree I strongly disagree

!_______ ! ______!_____ ________!_____________!

5 4 3 2 1

XIII. Do you consider that nurses have a professional duty as health professionals to offer to the caregivers’ education about patient care?

I strongly agree I agree neutral I disagree I strongly disagree

!_______ ! ______!_____ ________!_____________!

5 4 3 2 1

**8. INFORMATION NEEDS**

I. Do you consider it useful for the patients’ caregivers to receive information about the patient’s health status on a regular basis?

I strongly agree I agree neutral I disagree I strongly disagree

!_______ ! ______!_____ ________!_____________!

5 4 3 2 1

II. Do you consider it useful for the patients’ caregivers to receive information about monetary benefits provided by the insurance funds in order to buy rehabilitation products (wheelchair, crutches, prosthetic limbs, etc)?

I strongly agree I agree neutral I disagree I strongly disagree

!_______ ! ______!_____ ________!_____________!

5 4 3 2 1

III. Do you consider it useful for the patients’ caregivers to receive information about monetary benefits given by the insurance funds in order to hire a private helper?

I strongly agree I agree neutral I disagree I strongly disagree

!_______ ! ______!_____ ________!_____________!

5 4 3 2 1

IV. Do you consider it useful for the patients’ caregivers to receive information about where the patient could go after being discharged from the hospital, in case it is impossible to live at home?

I strongly agree I agree neutral I disagree I strongly disagree

!_______ ! ______!_____ ________!_____________!

5 4 3 2 1

V. Do you consider that the hospital has a responsibility to provide the patients’ caregivers with information on topics such as the above?

I strongly agree I agree neutral I disagree I strongly disagree

!_______ ! ______!_____ ________!_____________!

5 4 3 2 1

VI. Do you consider that providing information on topics such as the ones mentioned above, is a professional duty of nurses?

I strongly agree I agree neutral I disagree I strongly disagree

!_______ ! ______!_____ ________!_____________!

5 4 3 2 1

**QUESTIONNAIRE FOR NURSES**

**1. GENDER**

1. Male
2. Female

**2. AGE**

**3. MARITAL STATUS**

1. Single
2. Married
3. Widower- widow/Separated/Divorced

**4. EDUCATIONAL LEVEL**

1. Secondary education
2. University
3. Technological Educational Institute
4. Master studies
5. Master student
6. Phd
7. Phd student (candidate)

**5. WORK EXPERIENCE (YEARS)**

1. **<**10
2. 10-16
3. >16

**6.** **POSITION**

1. Head nurse or replacing the head nurse
2. Nurse
3. Assistant nurse /vocational nurse

**7. HEALTH EDUCATION NEEDS**

**Do you believe that it would be useful for the patients’ caregivers to be taught about:**

I. Techniques of patient care (for bathing, hair washing, changing position etc.)?

I strongly agree I agree neutral I disagree I strongly disagree

!_______ ! ______!_____ ________!_____________!

5 4 3 2 1

II. Basic knowledge about the patient’s disease when it is a chronic disease?

I strongly agree I agree neutral I disagree I strongly disagree

!_______ ! ______!_____ ________!_____________!

5 4 3 2 1

III. Prevention of pressure sores?

I strongly agree I agree neutral I disagree I strongly disagree

!_______ ! ______!_____ ________!_____________!

5 4 3 2 1

IV. How to manage complications in emergency situations that maybe the patient could develop in order to be able to provide first aid at home?

I strongly agree I agree neutral I disagree I strongly disagree

!_______ ! ______!_____ ________!_____________!

5 4 3 2 1

V. About the patient’s treatment at home in order to be able to help him/her at home?

I strongly agree I agree neutral I disagree I strongly disagree

!_______ ! ______!_____ ________!_____________!

5 4 3 2 1

VI. About the nutrition and daily fluid consumption that the patient should have at home?

I strongly agree I agree neutral I disagree I strongly disagree

!_______ ! ______!_____ ________!_____________!

5 4 3 2 1

VII. About how they can be effective when they seek help for the patient’s care by public services and N.G.O.s?

I strongly agree I agree neutral I disagree I strongly disagree

!_______ ! ______!_____ ________!_____________!

5 4 3 2 1

VIII. About how they can be effective when they seek help for the patient’s care by the extended family?

I strongly agree I agree neutral I disagree I strongly disagree

!_______ ! ______!_____ ________!_____________!

5 4 3 2 1

IX. About basic knowledge about the patient’s emotional needs and the possible changes in behavior that the disease might cause?

I strongly agree I agree neutral I disagree I strongly disagree

!_______ ! ______!_____ ________!_____________!

5 4 3 2 1

X. About how to deal with the stress that they might face due to their increased responsibility for the patient’s care?

I strongly agree I agree neutral I disagree I strongly disagree

!_______ ! ______!_____ ________!_____________!

5 4 3 2 1

XI. About (infection control) how to avoid spreading infections when they deal with the patient’s care?

I strongly agree I agree neutral I disagree I strongly disagree

!_______ ! ______!_____ ________!_____________!

5 4 3 2 1

XII. Do you consider that the hospitals have a responsibility to offer to the caregivers’ educational programmes about caring for patients with chronic health problems?

I strongly agree I agree neutral I disagree I strongly disagree

!_______ ! ______!_____ ________!_____________!

5 4 3 2 1

XIII. Do you consider that nurses have a professional duty as health professionals to offer to the caregivers’ education about patient care?

I strongly agree I agree neutral I disagree I strongly disagree

!_______ ! ______!_____ ________!_____________!

5 4 3 2 1

**8. INFORMATION NEEDS**

I. Do you consider it useful for the patients’ caregivers to receive information about the patient’s health status on a regular basis?

I strongly agree I agree neutral I disagree I strongly disagree

!_______ ! ______!_____ ________!_____________!

5 4 3 2 1

II. Do you consider it useful for the patients’ caregivers to receive information about monetary benefits provided by the insurance funds in order to buy rehabilitation products (wheelchair, crutches, prosthetic limbs, etc)?

I strongly agree I agree neutral I disagree I strongly disagree

!_______ ! ______!_____ ________!_____________!

5 4 3 2 1

III. Do you consider it useful for the patients’ caregivers to receive information about monetary benefits given by the insurance funds in order to hire a private helper?

I strongly agree I agree neutral I disagree I strongly disagree

!_______ ! ______!_____ ________!_____________!

5 4 3 2 1

IV. Do you consider it useful for the patients’ caregivers to receive information about where the patient could go after being discharged from the hospital, in case it is impossible to live at home?

I strongly agree I agree neutral I disagree I strongly disagree

!_______ ! ______!_____ ________!_____________!

5 4 3 2 1

V. Do you consider that the hospital has a responsibility to provide the patients’ caregivers with information on topics such as the above?

I strongly agree I agree neutral I disagree I strongly disagree

!_______ ! ______!_____ ________!_____________!

5 4 3 2 1

VI. Do you consider that providing information on topics such as the ones mentioned above, is a professional duty of nurses?

I strongly agree I agree neutral I disagree I strongly disagree

!_______ ! ______!_____ ________!_____________!

5 4 3 2 1
